# Periampullary and Pancreatic Metastases of Renal Cell Carcinoma: An Underdiagnosed Event

**DOI:** 10.14740/wjon911w

**Published:** 2015-06-12

**Authors:** Omar Y. Mousa, Rushikesh Shah, Nasser Hajar, Steve K. Landas

**Affiliations:** a Department of Medicine, State University of New York-Upstate Medical University, 750 E Adams St, Syracuse, NY 13210, USA; b Department of Gastroenterology, State University of New York-Upstate Medical University, 750 E Adams St, Syracuse, NY 13210, USA; c Department of Pathology, State University of New York-Upstate Medical University, 750 E Adams St, Syracuse, NY 13210, USA

**Keywords:** Renal cell carcinoma, Metastasis, Pancreatic, Periampullary, Mortality, Survival

## Abstract

The National Cancer Institute reports high incidence of renal cell carcinoma (RCC) in the US compared to other regions. However, pancreatic and periampullary metastasis are uncommon when only 17% of the RCC cases metastasize overall. We herein present a case series of four patients with periampullary or pancreatic metastatic disease following complete resection of RCC, evaluating their occurrences and outcomes. We reviewed the cases of four male patients retrospectively, mean age 75 years (range 65 - 87) who had a previous history of nephrectomy for RCC. They experienced recurrence with periampullary (two patients) or pancreatic (two patients) metastatic disease between 0 and 108 months (mean time 41.5 months) following primary tumor resection. In patients with periampullary metastasis, one had asymptomatic presentation with progressive dilatation of the pancreatic duct noted on surveillance CT scans. The other patient had iron deficiency anemia and melena with esophagogastroduodenoscopy (EGD) findings of large fungating infiltrative ulcerating mass in the area of the duodenal papilla (the only patient with metastasis to other sites: lungs and colon). As for those with pancreatic metastasis, one patient presented with hematuria and abdominal pain and was found to have pancreatic metastasis at the time of RCC diagnosis. The other patient was admitted for further workup of a mass in the pancreatic tail upon surveillance. Pathologic findings included high grade RCC in the metastatic foci. Management of such patients included: distal pancreatectomy in two patients without chemoradiation, one was awaiting Whipple procedure and received four cycles of sunitinib, while the last was a poor surgical candidate and received aminocaproic acid. Three patients are still alive to date. Optimal management is challenging given the very high risk of delayed relapse following tumor resection of the localized disease, leaving such cases with a very poor prognosis. Therefore to enhance survival, it is imperative to have careful stage-dependent surveillance in patients who have undergone a prior resection of RCC. We emphasize the importance of raising awareness for this unusual presentation. Disease recurrence as a pancreatic mass or hepatobiliary ductal dilatation might be more frequent than previously reported.

## Introduction

The National Cancer Institute reports high incidence of renal cell carcinoma (RCC) in the US compared to other regions [1]. RCC accounts for 2% of US cancer mortality, with an estimate of 13,000 deaths in 2008. The incidence continues to rise among all ethnic and age groups [2].

Even patients who had completed curative resection of the primary tumor had recurrence in one-third of the cases [3]. Pancreatic and periampullary metastases are uncommon among the 17% of RCCs that metastasize overall [4]. Such unusual metastases can have various clinical presentations, leading to a delayed diagnosis and higher mortality rates. Prognosis is poor with a median survival of 13 months [3]. Raising awareness among clinicians allow earlier identification and subsequent intervention with significant improvement in survival rates.

We present four cases with periampullary or pancreatic metastases following complete resection of RCC, evaluating their occurrences and outcomes compared to other cases in the literature.

## Case Report

We reviewed the case presentations of four male patients who had a previous history of nephrectomy for RCC, retrospectively. The mean age was 75 years (range 65 - 87 years). All four patients experienced recurrence with periampullary (two patients) or pancreatic (two patients) metastases from 0 to 108 months following primary RCC resection. The mean time of recurrence was 41.5 months.

The clinical presentations of the four patients (A, B, C and D) varied. Patient A with periampullary metastases had an asymptomatic presentation with progressive dilatation of the pancreatic duct noted on surveillance CT scans. He received four cycles of sunitinib followed by Whipple procedure.

Patient B had periampullary metastases as well but presented with melena and iron deficiency anemia with esophagogastroduodenoscopy (EGD) findings of large fungating infiltrative ulcerative mass in the area of duodenal papilla. This patient was the only one among the four with metastases to other sites: lungs and colon. He received aminocaproic acid and was later discharged home on palliative therapy given his extensive metastatic disease making him a poor surgical candidate.

Patient C had pancreatic metastasis at the time of RCC diagnosis and presented with hematuria and abdominal pain. He underwent distal pancreatectomy along with left radical nephrectomy and lymph node dissection.

Patient D was undergoing workup for a mass in the pancreatic tail that was found incidentally and the pathologic findings showed high grade RCC in the metastatic foci. This patient was managed with a distal pancreatectomy. Three patients are still alive to date.

## Discussion

Metastasis of primary RCC to the pancreas and periampullary region is uncommon. Very few cases with similar presentations to our case series were described in the literature. Although the lungs, bones, liver and brain are the most common sites of distant metastasis, patients with previous nephrectomies for primary RCC may develop delayed metastases to the pancreas and periampullary region. Table 1 below shows a comparison between data presented in the literature and the cases in our study, including the median times of occurrences of pancreatic or periampullary metastases. Overall, the median time of occurrence reported in the literature ranges from 6.5 to 12 years, with the longest interval being 32 years [5].

**Table 1:** Median Times of Occurrences of Pancreatic or Periampullary Metastases in Our Four Cases Compared to the Study Sample Presented by Villarreal-Garza et al

	Number		Gender		Age (mean/range)		Months from diagnosis to metastasis (range)		Surgical resection of metastasis		Status (alive)	
	Villarreal-Garza et al	Current study	Villarreal-Garza et al (M:F)	Current study (M:F)	Villarreal-Garza et al	Current study	Villarreal-Garza et al	Current study	Villarreal-Garza et al	Current study	Villarreal-Garza et al	Current study
Pancreas	11	2	7:4	2:0	55.6/42 - 68	77/67- 87	0 - 300	0 - 108	3	2	3/11	1/2
Duodenum/ampulla of Vater	4	2	3:1	2:0	50.8/43 - 59	73/65- 81	6 - 189	24 - 34	0	1	0/4	1/2

The 5-year survival rate in this patient population is < 10%. Resection of such metastases showed improved survival rates with a range from 43% to 88%. Even patients with multifocality of metastases from RCC experienced improved survival rates when complete resections were accomplished [5].

Optimal management is challenging given the very high risk of delayed relapse following tumor resection of the localized disease, leaving such cases with a very poor prognosis. In addition to an aggressive approach to detect early progression or recurrence, an individualized approach for surgical resection of metastases is significant for both palliation of symptoms and improved survival rates [5]. Therefore to enhance survival, it is imperative to have careful stage-dependent surveillance with prolonged follow-up in patients who have undergone a prior resection of RCC. A multicenter database review and analysis is needed to study the variable presentations and best approaches implemented to treat such rare metastases that have very poor prognosis. Such efforts can provide a better understanding of the patterns of pancreatic and periampullary metastases of RCC origin and help study new modalities for early detection and intervention and improved survival. We also emphasize the importance of raising awareness for this unusual presentation. Disease recurrence as a pancreatic mass or hepatobiliary ductal dilatation might be more frequent than previously reported.

## References

[1] Siegel R, Ma J, Zou Z, Jemal A (2014). Cancer statistics, 2014. CA Cancer J Clin.

[2] Chow WH, Devesa SS (2008). Contemporary epidemiology of renal cell cancer. Cancer J.

[3] Cohen HT, McGovern FJ (2005). Renal-cell carcinoma. N Engl J Med.

[4] SEER Stat Fact Sheets: Kidney and Renal Pelvis. http://seer.cancer.gov/statfacts/html/kidrp.html

[5] Villarreal-Garza C, Perez-Alvarez SI, Gonzalez-Espinoza IR, Leon-Rodriguez E (2010). Unusual Metastases in Renal Cell Carcinoma: A Single Institution Experience and Review of Literature. World J Oncol.

